# The *CACNA1B* R1389H variant is not associated with myoclonus-dystonia in a large European multicentric cohort

**DOI:** 10.1093/hmg/ddv255

**Published:** 2015-07-08

**Authors:** Niccolo E. Mencacci, Léa R'bibo, Sara Bandres-Ciga, Miryam Carecchio, Giovanna Zorzi, Nardo Nardocci, Barbara Garavaglia, Amit Batla, Kailash P. Bhatia, Alan M. Pittman, John Hardy, Anne Weissbach, Christine Klein, Thomas Gasser, Ebba Lohmann, Nicholas W. Wood

**Affiliations:** 1 Department of Molecular Neuroscience, Institute of Neurology, University College London, London WC1N 3BG, UK,; 2 Department of Physiology and Institute of Neurosciences Federico-Olóriz, Centro de Investigaciones Biomedicas (CIBM), University of Granada, Granada 18071, Spain,; 3 Neuropediatrics Unit, IRCCS Istituto Neurologico Carlo Besta, Milan 20133, Italy,; 4 Molecular Neurogenetics Unit, IRCCS Istituto Neurologico Carlo Besta, Milan 20133, Italy,; 5 Sobell Department of Motor Neuroscience and Movement Disorders, UCL Institute of Neurology, London WC1N 3BG, UK,; 6 Institute of Neurogenetics, University of Lübeck, Lübeck 23538, Germany and; 7 Department of Neurodegenerative Diseases, Hertie Institute for Clinical Brain Research, University of Tübingen, and German Center for Neurodegenerative Diseases (DZNE), Tübingen 72076, Germany

## Abstract

Myoclonus-dystonia (M-D) is a very rare movement disorder, caused in ∼30–50% of cases by mutations in *SGCE*. The *CACNA1B* variant c.4166G>A; (p.R1389H) was recently reported as the likely causative mutation in a single 3-generation Dutch pedigree with five subjects affected by a unique dominant M-D syndrome and cardiac arrhythmias. In an attempt to replicate this finding, we assessed by direct sequencing the frequency of *CACNA1B* c.4166G>A; (p.R1389H) in a cohort of 520 M-D cases, in which *SGCE* mutations had been previously excluded. A total of 146 cases (28%) had a positive family history of M-D. The frequency of the variant was also assessed in 489 neurologically healthy controls and in publicly available data sets of genetic variation (1000 Genomes, Exome Variant Server and Exome Aggregation Consortium). The variant was detected in a single sporadic case with M-D, but in none of the 146 probands with familial M-D. Overall, the variant was present at comparable frequencies in M-D cases (1 out of 520; 0.19%) and healthy controls (1 out of 489; 0.2%). A similar frequency of the variant was also reported in all publicly available databases. These results do not support a causal association between the *CACNA1B* c.4166G>A; (p.R1389H) variant and M-D.

## Introduction

Myoclonus-dystonia [M-D (MIM 159900)] is a rare familial movement disorder, which classically features a variable combination of non-epileptic myoclonic jerks and dystonia (1). Heterozygous loss-of-function mutations in the maternally imprinted ε-sarcoglycan gene [*SCGE*, DYT11; (MIM 604149)] represent a major cause of autosomal dominant M-D (2). However, up to 50–70% of familial cases with M-D lack mutations in *SGCE* (3–5), suggesting that disease-causing mutations in other genes are responsible for this syndrome.

Recently, Groen *et al*. (6) identified the missense variant c.4166G>A; (p.R1389H) (rs184841813) in *CACNA1B* [MIM 601012] as the likely causative mutation in a Dutch pedigree with five subjects affected by autosomal dominant M-D lacking mutations in *SGCE*. Unique features in the pedigree were lower limb orthostatic high-frequency myoclonus, attacks of limb painful cramps and cardiac arrhythmias in three of the affected subjects (7). Sanger sequencing of the *CACNA1B* exons coding for the protein portion spanning from III-S5 to III-S6 failed to reveal other mutations in a further 47 M-D cases.

*CACNA1B* encodes neuronal voltage-gated calcium channels CaV2.2, which have a key role in controlling synaptic neurotransmitter release (8). Furthermore, *CACNA1A* [MIM 601011] mutations in the homologous region of the gene cause familial hemiplegic migraine [MIM 141500] (9) and episodic ataxia type 2 [MIM 108500] (10).

The *CACNA1B* p.(R1389H) substitution represents therefore an excellent candidate as a disease-causing mutation for M-D. However, in the absence of identification of *CACNA1B* mutations in other unrelated pedigrees, the implication of mutations in this gene as a cause for M-D is not confirmed.

In this study, we assessed the frequency of the *CACNA1B* c.4166G>A; (p.R1389H) variant in a large multicentric cohort of M-D cases without mutations in *SGCE* (both point mutations and copy number variants).

## Results

A total of 520 M-D cases (28% were familial) were screened for the presence of the c.4166G>A; (p.R1389H) variant. Additionally, we assessed the frequency of the variant in whole-exome sequencing data from 489 white healthy controls of UK and US origin and in European cases listed in publicly available data sets of genetic variation (1000 Genomes, Exome Variant Server and Exome Aggregation Consortium).

None of the 146 probands with familial M-D carried the *CACNA1B* c.4166G>A; (p.R1389H) variant. The variant was detected only in a single female case of UK origin with sporadic M-D (see chromatogram of the mutation in the Supplementary Material, Fig. S1). This case presented in her mid-30s with tremulous cervical dystonia and myoclonic jerks in the upper limbs. She had no family history for M-D or any other movement disorder. No other family members were available for segregation analysis of the variant.

The total carrier frequency in our M-D cohort, including familial and sporadic cases, is 0.19% (1/520 cases). The variant is present at a similar frequency in our healthy controls (0.2%; 1 out of 489 individuals). The control carrier of the variant is a 38-year-old male without any neurological disease and with no relevant family history of movement disorders.

The *CACNA1B* c.4166G>A; (p.R1389H) variant is reported at comparable frequencies in the 1000 genome project (0.26%; 1/379 individuals) and Exome Variant Server (0.28%; 12/4203 individuals) databases. In the Exome Aggregation Consortium database, c.4166G>A; (p.R1389H) is present in 0.11% (38 out of 33 367) of the European subjects (difference to M-D cases not significant; Fisher's exact test *P* = 0.4).

## Discussion

The advent of next generation sequencing has led to an extraordinary acceleration in the discovery rate of rare genetic variants, the majority of which are of uncertain clinical significance. Hence, a close scrutiny is necessary before causally linking a candidate variant to a disease. To avoid false assignment of pathogenicity, MacArthur *et al*. (11) have recently proposed guidelines for implicating causality of rare variants in human disease.

In family-based studies, assessment of co-inheritance of a candidate variant with the disease status within family members represents the first requirement to prove causality.

The c.4166G>A; (p.R1389H) variant was identified by Groen et al. through a combination of whole-exome sequencing and linkage analysis (13 chromosomal regions identified, with a maximum LOD score of 1.2) in a single dominant M-D pedigree. Notably, two other rare missense changes, c.10355A>G; (p.Q3452R) in *VPS13D* [MIM 608 877] and c.5308C>T; (p.R1770C) in *SPTAN1* [MIM 182810], were found to perfectly co-segregate with the disease in the family. *De novo* mutations in *SPTAN1* have been shown to cause a neurological phenotype (West syndrome with severe cerebral hypomyelination, spastic quadriplegia and developmental delay) (12) and more recently a microdeletion encompassing *SPTAN1* was detected in a child with epileptic encephalopathy and severe dystonia (13).

Given the clinical presentation pointing towards a possible channelopathy, the authors assumed that the causative variant was the one in *CACNA1B* (6).

However, co-segregation of a variant with disease in a single pedigree does not establish with certainty its pathogenic role, especially if other co-segregating coding variants and the possibility of a separate undetected pathogenic variant in linkage disequilibrium cannot be convincingly ruled out.

In addition, a candidate variant responsible for a rare disease should be found at a low frequency in population controls, consistent with the proposed model of inheritance and disease prevalence.

M-D is a very rare disorder with a suggested prevalence of around two per million in Europe (14). We would therefore anticipate highly penetrant mutations causing dominant forms of M-D to be absent or extremely rare in the general population. Yet, this is not the case for p.(R1389H), which is present at a considerable frequency in our healthy controls and all publicly available databases (∼0.1–0.3%). According to the Exome Aggregation Consortium database, the carrier frequency of this variant in Europeans is ∼4 times higher than the *TOR1A* [MIM 605204] c.904_906delGAG deletion (0.026%), which is by far the most common single mutation responsible for dystonia described to date (15). Given this frequency, if c.4166G>A; (p.R1389H) were a pathogenic variant, we would expect it to be responsible for a large proportion of familial M-D cases. However, in our cohort, not only was the variant not identified in any of the probands with familial M-D, but the overall frequency of the variant did not differ between M-D cases and healthy controls. This does not support a pathogenic effect of the variant even assuming a reduced penetrance.

In conclusion, our study suggests that the role of the *CACNA1B* variant c.4166G>A; (p.R1389H) as a cause for M-D is questionable. Further genetic evidence is needed before designating *CACNA1B* mutations as a cause for dominant M-D.

## Materials and Methods

A total of 520 M-D cases of British, German and Italian origin were recruited in four tertiary movement disorders centers (London, Lübeck, Tübingen and Milan). All selected cases fulfilled the proposed diagnostic criteria for M-D (2). A total of 146 cases (28%) had a positive family history of M-D. All participants provided written informed consent.

M-D cases were screened by direct Sanger sequencing for mutations in exon 28 of *CACNA1B* (RefSeq NM_000718.3), which contains the c.4166G>A; (p.R1389H) variant. Each reaction was performed in a 20 µl volume containing 10 µl of FastStart PCR master mix (Roche), 5 µl of water, 2 µl of each primer (5pmol/µl) and 30 ng of genomic DNA. After purification, PCR products were sequenced in both forward and reverse directions using BigDye Terminator v3.1 sequencing chemistry and then were loaded on the ABI3730xl genetic analyser (Applied Biosystems, Foster City, CA, USA). The sequences were analysed with Sequencher software (version 4.9; Gene Codes).

Whole-exome sequencing data from 489 white healthy controls of UK and US origin were provided by the International Parkinson's Disease Genomic Consortium (IPDGC). In short, prior to sequencing, DNA templates were bridge amplified to form clonal clusters inside a flowcell via the cBot cluster generation process. The flowcells were then loaded into the next-generation sequencer Illumina HiSeq 2000. Paired end sequence reads were aligned with Burrows-Wheeler Aligner (BWA) against the reference human genome (UCSC hg19). Duplicate read removal, format conversion and indexing were performed with Picard (http://picard.sourceforge.net/). The Genome Analysis Toolkit (GATK) was used to recalibrate base quality scores, perform local realignments around possible indels and to call and filter the variants.

## Web Resources

1000 Genomes project (URL: http://www.1000genomes.org/) [last accessed: April 2015].

Exome Variant Server, NHLBI GO Exome Sequencing Project (ESP), Seattle, WA, USA (URL: http://evs.gs.washington.edu/EVS/) [last accessed: April 2015]

Exome Aggregation Consortium (ExAC), Cambridge, MA, USA (URL: http://exac.broadinstitute.org) [last accessed: April 2015].

## Supplementary material

Supplementary Material is available at *HMG* online.

## Funding

The work was undertaken at University College London Hospitals (UCLH) and University College London (UCL), who receive support from the Department of Health's NIHR Biomedical Research Centers funding streams. N.E.M. is funded by an MRC-Wellcome Trust grant. S.B. holds an FPU fellowship from the Spanish Ministry of Education and Science jointly with a short-term stay grant by Cei-BioTic and University of Granada. A.M.P. is funded by the Reta Lila Weston Trust. C.K. is the recipient of a career development award from the Herman and Lilly Schilling Foundation. E.L. and T.G. are supported by a grant from the Dystonia Medical Research Foundation (DMRF). The funders had no role in study design, data collection and analysis, decision to publish or preparation of the manuscript. This work was supported by a Medical Research Council/Wellcome Trust Strategic Award (grant number WT089698/Z/09/Z) and a grant from the Bachman-Strauss Dystonia Parkinsonism Foundation. Funding to pay the Open Access publication charges for this article was provided by Medical Research Council/Wellcome Trust

## Supplementary Material

Supplementary Data
