# Supplementary material for: The CACNA1B R1389H variant is not associated with myoclonus-dystonia in a large European multicentric cohort
Source: Hum Mol Genet. 2015 Jul 8;24(18):5326–9. doi: 10.1093/hmg/ddv255 (PMC4550822; doi:10.1093/hmg/ddv255)
Supplement: Supplementary Data [file supp_24_18_5326__index.html]

The CACNA1B R1389H variant is not associated with myoclonus-dystonia in a large European multicentric cohort — The CACNA1B R1389H variant is not associated with myoclonus-dystonia in a large European multicentric cohort — The CACNA1B R1389H variant is not associated with myoclonus-dystonia in a large European multicentric cohort — Supplementary Data 

# The *CACNA1B* R1389H variant is not associated with myoclonus-dystonia in a large European multicentric cohort

## Supplementary Data

Supplementary Data

- Supplementary Data - Docx file
