# Supplementary figures and images for: The CACNA1B R1389H variant is not associated with myoclonus-dystonia in a large European multicentric cohort
Source: Hum Mol Genet. 2015 Jul 8;24(18):5326–9. doi: 10.1093/hmg/ddv255 (PMC4550822; doi:10.1093/hmg/ddv255)

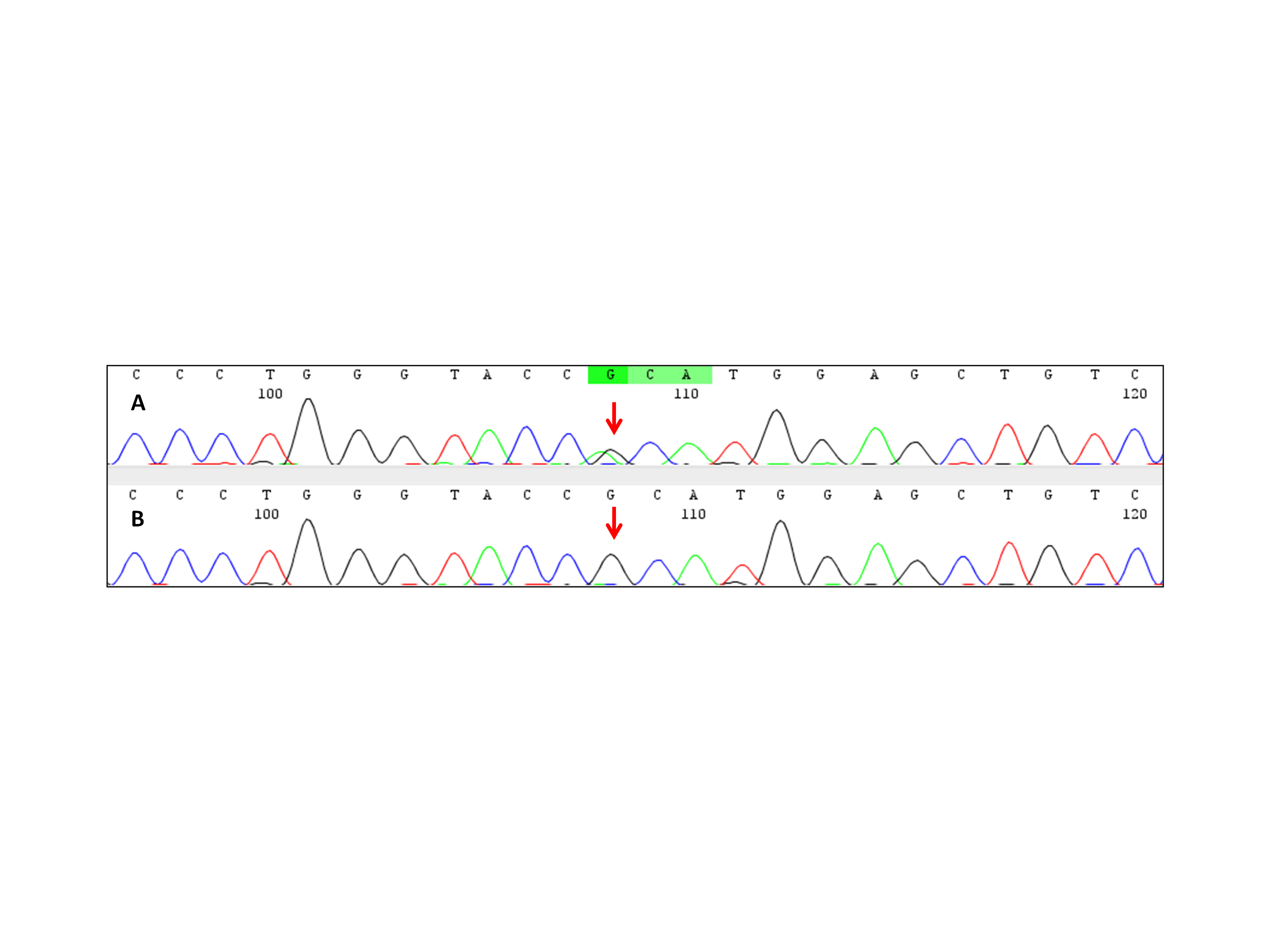


Figure S1. Chromatograms showing the *CACNA1B* c.4166G>A; (p.R1389H) variant (A) and a control sequence (B).

Supplement: Supplementary Data [file supp_ddv255_ddv255supp.docx]
